# A translational perspective towards clinical AI fairness

**DOI:** 10.1038/s41746-023-00918-4

**Published:** 2023-09-14

**Authors:** Mingxuan Liu, Yilin Ning, Salinelat Teixayavong, Mayli Mertens, Jie Xu, Daniel Shu Wei Ting, Lionel Tim-Ee Cheng, Jasmine Chiat Ling Ong, Zhen Ling Teo, Ting Fang Tan, Narrendar RaviChandran, Fei Wang, Leo Anthony Celi, Marcus Eng Hock Ong, Nan Liu

**Affiliations:** 1 Centre for Quantitative Medicine, Duke-NUS Medical School, Singapore, Singapore; 2 Centre for Ethics, Department of Philosophy, University of Antwerp, Antwerp, Belgium; 3 Antwerp Center on Responsible AI, University of Antwerp, Antwerp, Belgium; 4 Department of Health Outcomes and Biomedical Informatics, University of Florida, Gainesville, FL USA; 5 Singapore Eye Research Institute, Singapore National Eye Centre, Singapore, Singapore; 6 SingHealth AI Office, Singapore Health Services, Singapore, Singapore; 7 Department of Diagnostic Radiology, Singapore General Hospital, Singapore, Singapore; 8 Department of Pharmacy, Singapore General Hospital, Singapore, Singapore; 9 Department of Population Health Sciences, Weill Cornell Medicine, New York, NY USA; 10 Laboratory for Computational Physiology, Massachusetts Institute of Technology, Cambridge, MA USA; 11 Division of Pulmonary, Critical Care and Sleep Medicine, Beth Israel Deaconess Medical Center, Boston, MA USA; 12 Department of Biostatistics, Harvard T.H. Chan School of Public Health, Boston, MA USA; 13 Programme in Health Services and Systems Research, Duke-NUS Medical School, Singapore, Singapore; 14 Department of Emergency Medicine, Singapore General Hospital, Singapore, Singapore; 15 Institute of Data Science, National University of Singapore, Singapore, Singapore

**Keywords:** Medical research, Health care

## Abstract

Artificial intelligence (AI) has demonstrated the ability to extract insights from data, but the fairness of such data-driven insights remains a concern in high-stakes fields. Despite extensive developments, issues of AI fairness in clinical contexts have not been adequately addressed. A fair model is normally expected to perform equally across subgroups defined by sensitive variables (e.g., age, gender/sex, race/ethnicity, socio-economic status, etc.). Various fairness measurements have been developed to detect differences between subgroups as evidence of bias, and bias mitigation methods are designed to reduce the differences detected. This perspective of fairness, however, is misaligned with some key considerations in clinical contexts. The set of sensitive variables used in healthcare applications must be carefully examined for relevance and justified by clear clinical motivations. In addition, clinical AI fairness should closely investigate the ethical implications of fairness measurements (e.g., potential conflicts between group- and individual-level fairness) to select suitable and objective metrics. Generally defining AI fairness as “equality” is not necessarily reasonable in clinical settings, as differences may have clinical justifications and do not indicate biases. Instead, “equity” would be an appropriate objective of clinical AI fairness. Moreover, clinical feedback is essential to developing fair and well-performing AI models, and efforts should be made to actively involve clinicians in the process. The adaptation of AI fairness towards healthcare is not self-evident due to misalignments between technical developments and clinical considerations. Multidisciplinary collaboration between AI researchers, clinicians, and ethicists is necessary to bridge the gap and translate AI fairness into real-life benefits.

## Introduction

The early days of artificial intelligence (AI) were filled with great aspirations, some of which have now been realized, particularly in the “post-ChatGPT” era^1–4^. In healthcare, data-driven AI models have shown capability in extracting objective evidence from complex and large-scale databases^5,6^. Yet, algorithms are only as objective as the data that they are based on. Similarly, human judgments are inevitably susceptible to bias in handling sensitive data (e.g., age, gender/sex, race/ethnicity, socio-economic status, weight, sexual orientation) even when these data variables have no objective connection with the outcome of interest^7^. In high-stakes fields like clinical decision-making, fairness (or absence of bias) is of vital importance. Proper application of AI fairness in clinical algorithmic development could contribute to the reduction of health disparities rather than their escalation^8,9^ but practical implementation is not self-evident.

The practice of medicine has continuously been evolving from eminence-based to evidence-based, but due to limited resources, the evidence may be gathered from a skewed representation of the underlying population, e.g., in terms of race/ethnicity or age subgroups. The emerging data-driven practice in medical decision-making may reduce the risk of bias, but if not carefully designed, decision rules generated can still lead to unfair decisions^10^. For example, the online Kidney Donor Profile Index (KDPI) calculator used by the US Organ Procurement & Transplantation Network predicts higher risks of kidney graft failure for black donors than for non-black donors when all other conditions are identical, resulting in fewer eligible organ sources from black donors^11^.

Such risk of bias is not automatically mitigated by using more complex algorithms or a larger amount of data^12,13^. In one example, questionable differences are observed in AI-based survival prediction after liver transplantation by gender^14^, which can bias clinical decisions and allocation of scarce healthcare resources against certain patient subgroup(s) simply because of the traits they were born with. Such biased models violate the justice required in delivering equal well-being in healthcare. It is essential to develop fair models for data-driven clinical decision-making, but current AI fairness research may not be well-adaptable for clinical settings.

**Fig. 1:**
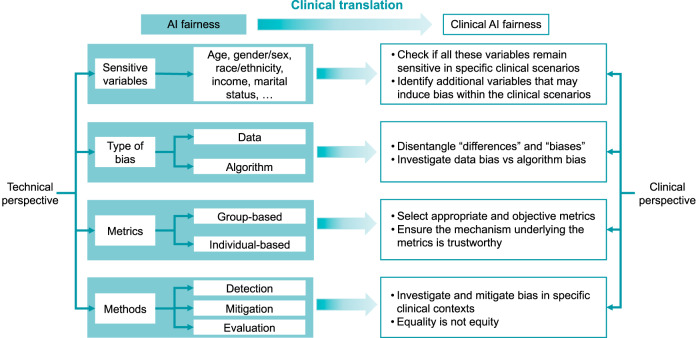
Conceptual model towards clinical AI fairness. Left panel: the framework of AI fairness from the technical perspective; right panel: the corresponding concerns from the clinical perspective, which are not yet (fully) addressed by current methodological developments.

In recent years, with growing public awareness of bias in AI models in real-life tasks such as face recognition^15^ and prediction of recidivism^16^, AI researchers have developed extensive qualitative and quantitative approaches to evaluate and ensure fairness in model development^17,18^. However, due to the knowledge gap amongst AI researchers and clinicians, AI fairness studies tend to focus on abstractive conceptualization or technical developments. While these aspects are highly important, it is unclear how they can be applied to healthcare. This paper provides an overview of the misalignments of current AI fairness research with practical clinical concerns and the obstacles to AI fairness adaptation, which is visually summarised in Fig. 1.

## AI fairness from a technical perspective

Fair AI has been associated with a variety of technical properties and capabilities. It is widely believed that AI is capable of making accurate predictions. Additionally, AI is expected to remain robust against the cognitive bias and prejudice that humans experience when making judgments, and even to detect biases that humans cannot recognize^17,18^. This builds on a series of concepts and methods, as visually summarized under “AI fairness” in Fig. 1 and elaborated below.

### Bias and fairness types

Bias and fairness are two concepts that usually oppose each other: a decision is unfair if it is biased towards (or against) any individual or subpopulation^19^. In the development pipeline of an AI model, which typically involves data collection, model training, evaluation, and validation, bias (and therefore unfairness) can occur at any stage for various reasons, sometimes in an imperceptible manner^20^.

First, any historical (and existing) bias in medical practice can be reflected in medical records, e.g., underdiagnosis and undertreatment of postpartum depression has been observed among minorities on Medicaid^21,22^, which will bias the resulting prediction models in similar ways if not carefully handled. Data under-representation^23^ is another common source of data bias that arises from inappropriate data collection and sampling, where certain subgroups constitute a smaller proportion of the sample than they are in the underlying population, leading to biased inference and predictions. For example, the landmark Framingham Heart Study greatly improved the understanding of cardiovascular disease but was more beneficial to Caucasians than to other underrepresented ethnic groups in the USA^24^. Data bias may be amplified by inappropriate data pre-processing, including but not limited to the exclusion of incomplete records when information is not missing at random, or a naive combination of datasets from different sources without accounting for overlapping subjects. All possible sources of data bias should be proactively identified and addressed during the early stages of AI model development before it impedes fair model development.

In addition to data bias, inappropriate model development steps (e.g., unjustifiable use of sensitive variables such as gender/sex and race/ethnicity in decision-making) can amplify existing bias or introduce new bias in AI models, resulting in algorithm bias that is another prevalent source of AI unfairness^9^. The use of black-box AI models, especially complex deep learning models, exacerbates algorithm bias by making it more difficult to detect and understand. Algorithm bias can be mitigated, but often at the expense of model performance^25^, for example, when intentionally excluding sensitive variables that can add information for outcome prediction in the development data. This makes it difficult to develop and implement completely fair AI in pragmatic healthcare practice.

### Fairness metrics in AI literature

Many quantitative metrics have been developed to assess fairness in AI, mostly from the perspective of “equality”^17,18,26^: a fair model should have equal performance in subgroups with respect to sensitive variables. Two types of fairness metrics are discussed most often: group-based and individual-based^17,18,26^. Group-based metrics measure the consistency of model performance (e.g., using the confusion matrix or calibration) across subgroups defined by sensitive variables, and a fair model is expected to behave similarly among subgroups. Some widely used individual-based metrics include fairness through awareness^27^ which assumes that observations with similar conditions should have similar predictions, and counterfactual fairness^28^ expecting that changing a sensitive variable should not alter the predicted outcome for an individual. Interested readers can refer to Supplementary Table 1 for a more detailed overview of fairness metrics.

### Methods to detect, prevent, and mitigate bias

To ensure the fairness of AI models, each step of the modeling pipeline should be self-motivated and aware of fairness^26^, even for data exploration^29^. A detailed description of datasets (e.g., time-period and site information for data collection) can provide evidence to detect data bias such as under-representation of any subpopulation^23^ for early bias prevention. A simple way to resolve such data bias is to collect or request additional data, but this is not always feasible due to regulations and legislation. In this case, AI researchers can pre-process existing data using appropriate sampling methods to better represent the underlying population^30^, and use regular methods to develop models from the adjusted dataset. Some prototype methods are listed in Supplementary Table 2 as examples.

In addition to the pre-process approach described above, there is a rich body of research on methods to mitigate data bias in- or post-process during or after model development, respectively, using the fairness metrics described in the previous subsection as bias-monitoring and fairness-evaluation tools. Typical in-process methods include adding fairness constraints served by fairness metrics, and representation learning by filtering the sensitive information for decision-making, whereas post-process methods primarily rely on catering the established model for sensitive subgroups (see Supplementary Table 2 for examples).

## AI fairness from a clinical perspective

The AI fairness technologies described in the previous section have been applied in healthcare research, yet there remains a prominent gap in the understanding of “fairness” between AI developers and healthcare providers. The part of Fig. 1 under “Clinical AI fairness” lists examples of important considerations in clinical AI fairness not yet (fully) addressed by current methodological developments, which may explain the limited adoption of AI fairness in clinical applications. In this section, we summarize the potential hurdles and challenges in AI fairness in healthcare, in order to promote future applications in clinical settings.

### Hurdles for evaluating fairness in healthcare

On top of AI fairness metrics discussed in the previous section, the mechanisms behind the fairness metrics can be problematic from the perspective of healthcare. For example, the theoretically well-defined and well-received counterfactual fairness^28^ assumes that the prediction should remain unchanged for an individual when changing the value of a sensitive variable (e.g., female to male) with all other variables unchanged. This may be plausible when predicting the likelihood of being hired by a company, but less so in clinical contexts with natural biological differences between females and males^31^, where artificially changing one sensitive variable while leaving others unchanged may lead to comparison with a “phantom” improbable to exist in real life.

Secondly, different types of fairness metrics correspond to varying fairness definitions, which may in some cases conflict in perspectives and ethical principles: group-based fairness may be more relevant to the perspective of hospital leadership or public health policy-making on the basis of population ethics, whereas individual-based metrics are closer to the perspective of patient-level decision-making guided by clinical ethics^32^. Such differences in ethical assumptions and clinical perspectives should be accounted for and justified when applying fairness metrics in healthcare applications, and failing to account for either individual- or group-based fairness seems unethical^33^.

The choice of fairness metrics is further complicated by the large number of metrics available that may produce inconsistent results^26^. Though there have been several review papers discussing the relationships and differences between these metrics, they do not provide practical guidelines regarding the selection of fairness metrics to address specific clinical needs^34^. Due to the trade-offs between the metrics, it is mathematically impossible to optimize all metrics simultaneously, except in highly restrictive cases^26,33,35^.

Moreover, group-based metrics are “secondary” metrics, which reflect differences in primary performance metrics across subgroups^17,18,26,36^, such as the commonly applied fairness metric — equality of opportunity defined as the differences in true positive rates among subgroups^37^; however, objective thresholds are desperately needed to differentiate reasonable differences from evidence of bias. Several hypothesis testing methods^38,39^ have been proposed to statistically assess the presence of a difference, but when the sample size is sufficiently large, small differences can appear statistically significant even when it is clinically non-significant^40^. It would be relevant to incorporate such considerations when modifying existing fairness metrics or devising new ones for clinical AI models to avoid misclaims of fairness.

### Differences or biases?

As discussed in the previous section, “differences” are roughly equivalent to “biases” in general AI fairness research in bias detection and fairness evaluation, where most biases are claimed based on “secondary” metrics derived from differences of primary performance metrics^30,41^. However, these two terms are distinct, where “differences” refers to variations among individuals or groups and requires respect, while “bias” refers to unfair preferences or prejudices towards certain individuals or groups and requires mitigation^42^. When coming to the clinical context, differences and biases can be difficult to disentangle, and failure to distinguish them could lead to negative consequences^43^. On the one hand, claims of differences can be biased if they lack solid justification; for instance, genetic differences between race/ethnicity subgroups in relation to certain diseases can be controversial^10^, so as the resulting differences detected by fairness metrics. In addition, when the biomedical differences have been identified, e.g., males hold a higher risk of non-small-cell lung cancer than females^44^, bias detection remains a challenge, as it is difficult to assess if the differences observed are fully justifiable by biomedical reasons or are partially due to unknown bias.

On the other hand, simplistic claims of biased predictions could conceal the real problems that merit further investigations. After reporting bias in models, most studies either stopped there or tried to mitigate the bias via model adjustments. However, forced adjustments for equal performance across subgroups may impair model stability and limit generalizability^45^. More importantly, when studying adverse outcomes such forced performance improvement for under-privileged subgroups to some extent approves existing unfairness and justifies existing health disparities rather than reduces them. In such cases, underpinning the cause of disparity to enable subsequent interventions is practically more desirable. As an example, breast cancer studies in Singaporean cohorts reported worse outcomes for Malaysian females than for other ethnicity groups. In-depth investigations revealed that Malaysian females were more hesitant to seek medical examinations and treatments due to cultural reasons, causing delayed diagnosis and hence worse clinical outcomes^46^. Such findings provide hints for possible interventions to improve real-life health outcomes.

### The problematic assumption underlying current fair AI methodologies

Superficially considering difference as bias, current methodologies of AI fairness mainly contribute to solving clinical questions that particularly assume “equality” as evidence of non-bias (fairness)^17,18^, such as equal chances of receiving treatment among pre-defined subgroups (e.g., by age or gender). Despite the fact that this assumption of “equality” is practical in quantifying fairness as an abstractive concept, which led to its widespread adoption in general AI fairness, it is normally embedded with a strong implicit assumption that the treatment is equally suitable and hence must be made equally available for all subgroups if all other conditions are identical. Such an assumption is clearly irrational for some sensitive variables, such as age, which is an important consideration in any clinical decision-making. Moreover, insisting on equality in treatment regardless of patients’ age and the corresponding prognosis neglects important dimensions of medical practice including dignity preservation and quality of life optimization. Thus, focussing on principles of equity rather than equality may push concepts of fairness beyond the common discussion in general fair AI research. This will likely require the inclusion of contextual factors such as patients’ preferences. Moreover, definitions of AI fairness must be contextualized to clinical and social scenarios, which would inevitably involve different sets of assumptions, and be informed by real-life feasibility.

### Rethinking “sensitive variables” with respect to healthcare scenarios

In general fair AI studies, sensitive variables such as age, gender/sex, race/ethnicity, social status, marital status, and disability status are repeatedly mentioned and a fair decision-making process is expected to be free from the influence of such information^17,47^. However, as discussed above, some of these variables are highly relevant to disease diagnosis, treatment decisions and prognosis, and therefore cannot be hidden from clinical decision-making and healthcare resource allocation.

When developing fair AI for clinical outcomes, the set of relevant sensitive variables should be carefully re-evaluated for each application with clinical justifications. Examining the role of sensitive variables in clinical decision-making presents a significant challenge, as it requires a case-by-case analysis. This may be done by investigating the relationship between sensitive variables and the outcome (e.g., the presence of correlation or causation), and accordingly exclude or include the sensitive variables to facilitate fair decision-making in the specific context, with an objective of “equality” or “equity” as appropriate. Race/ethnicity is particularly challenging to handle in the pursuit of “equity”, as it can be associated with systemic bias that affects clinical practice, or genuine biological and/or sociological differences among subpopulations^48^. The aforementioned postpartum depression study is a typical example of systemic racial/ethnic bias inducing a correlation between this sensitive variable with the outcome, which needed to be corrected by including this variable with additional bias mitigation procedures instead of simply excluding racial information^21,22^. Whether an observed race/ethnicity-related difference is genuine and justifiable can be controversial and requires additional investigations. For example, while the KDPI score to predict kidney graft failure was criticized for predicting a higher risk for black donors, further investigations revealed that this may be justified by differences in some genetic factors^49^. Hence, instead of using race/ethnicity as an easy surrogate, it is preferable to replace the proxy with the underlying factors (in this example the genetic factors) that have a causal effect on the outcome^24,50^. Such follow-up studies can also help identify modifiable factors to improve healthcare outcomes, instead of passively associating inferior outcomes with some racial subgroups.

When tailoring AI fairness to clinical outcomes, researchers also need to reframe the concept of “fairness” in the specific clinical context, and a fair treatment decision requires other crucial considerations that are not applicable to general AI fairness research. These considerations include factors that may induce over-treatment (over-diagnosis) or under-treatment (under-diagnosis)^21,51^, confounders for treatment effects^52^, patients’ implicit considerations of interests such as end-of-life care preferences, clinicians’/patients’ prejudice towards a specific treatment, and lack of AI digital literacy that may limit lower-resource communities from adopting and benefitting from AI, etc. These questions are currently overshadowed by traditionally recognized sensitive variables, hindering the applications of well-established methodologies in fair AI.

### Clinicians in the loop with fair AI

Clinical AI fairness is a multidisciplinary research topic that requires input from AI researchers, clinicians, and ethicists. Some of the concerns regarding clinical AI fairness have already been discussed and addressed in medical ethics, bioethics and epidemiological literature^43,53–55^, calling for effective cross-disciplinary communication. Engaging multidisciplinary experts in active discussions can enhance existing AI governance schemes to promote fairness. Measures such as the establishment of data panels to oversee the data collection and avoid data bias^56^, dynamic monitoring of model fairness in adherence to ethical principles within healthcare workflows^57^, and incorporation of fairness considerations into clinical AI guidelines^58^ contribute to raising awareness and enhance the fairness of AI applications in healthcare.

In addition to the role of governance, clinicians can contribute more proactively to the multiple stages of fair AI model development. Despite the growing awareness of the potential of AI models in medical research^5,6^, clinicians typically only participate in AI modeling in limited ways, such as providing general context description and validating the alignment of predictions and actual decisions, where they are frequently out of the modeling process^5^. However, clinicians possess the ability to not only proactively identify potential biases within specific clinical context for earlier bias detection, but also to discern bias and clinically meaningful differences to set appropriate objectives for AI models^21,22^. In addition, with clinicians evaluating the algorithms purportedly addressing bias, standard clinical significance regarding fairness can be put forward under clinical common sense. Thereafter, only the models with clinically significant bias should be adjusted, avoiding over-adjustment or over-claim of bias.

To align the objectives of AI developers and clinicians, it is necessary to establish a two-way communication between the two parties to facilitate an iterative model building process that aligns technical rationale with clinical concerns. For example, fairness metrics applied (or developed) by AI developers should be clinically contextualized to accurately quantify fairness in clinical settings. Such communication requires some functional understanding of AI modeling for clinicians^59^ and clinical context grasping for AI developers^56^. Explainable AI can contribute to such communication since it provides clinicians with the capability of interpreting models^60^ and giving feedback to the AI developers^61^. Having a better understanding of the model’s decision-making process could enable clinicians to help improve the model’s accuracy, and clinicians would also guide the algorithms in a more equitable direction.

## Conclusions

Current AI fairness research may not be readily adaptable to clinical settings. With the discussion of multiple misalignments between the technical and clinical perspectives, we highlighted the obstacles to clinical AI fairness translation, which requires multidisciplinary collaboration among clinicians, AI researchers, social scientists, philosophers, and beyond.

### Supplementary information

Supplementary tables
