# Supplementary material for: A translational perspective towards clinical AI fairness
Source: NPJ Digit Med. 2023 Sep 14;6:172. doi: 10.1038/s41746-023-00918-4 (PMC10502051; doi:10.1038/s41746-023-00918-4)
Supplement: Supplementary file 1 — Supplementary tables [file 41746_2023_918_MOESM1_ESM.pdf]

Supplementary Table 1. Fairness definition and metrics for evaluation.

Fairness definition	Metrics category	Description	Prototype metrics
Group fairness: To dictate an equal outcome probability between subgroups separated by a sensitive variable	Parity-based	Focusing on predicted positive values	Demographic (statistical) parity[1], disparate impact[2], conditional statistical parity[1]
	Performance-based	1) Addressing the equality of performance metrics (e.g., accuracy, sensitivity, etc.) among different subgroups<br>2) Focusing on the calibration between average predicted probability and fractions of positive values	Equal opportunity[3], equalized odds[3,4], other metrics computed by differences or ratios of machine learning metrics among subgroups (e.g., accuracy equality[5])
	Rank-based	Focusing on the relative ranking of scores among outcome classes (e.g., mortality and survival), expected to be independent on sensitive groups	Disparity in bipartite-ranking metrics (e.g., ΔxAUC[6])
Individual fairness: To encode the notion that comparable individuals should be treated equally	Similarity-based	Emphasizing similar results from similar individuals	Fairness with awareness[7]
	Counterfactual-based	Emphasizing unchanged results after changing the sensitive information	Counterfactual fairness[8]
Distributive fairness: To address fair distribution of limited resources (e.g., vaccination) across multiple stakeholders[9,10]	Variance-based	Emphasizing the equality of quantities received by participants via minimizing variation	Variance or standardized deviation of the quantities[11] (e.g., accuracy, loss, etc.)
	Reward-based	Emphasizing alignments between the quantities received by participants and their inputs and efforts	Reward based on correlation[12]

Supplementary Table 2. Method to mitigate bias and strive for fairness.

Methods category	Description	Prototype methods
Pre-process	To remove bias in the data	Resampling[13,14], reweighting[13,15]
In-process	To build a fair model, with original data that may or may not have bias	Representation learning[16] (e.g., adversarial learning[17]), prejudice remover[18,19], subgroup modelling[20]
Post-process	To fine-tune the model according to different subpopulations	Equalized odds post-processing[3], adjusting thresholds for subgroups[21], fine-tune the model according to subgroups[22]

Supplementary References

1 Corbett-Davies, S., Pierson, E., Feller, A., Goel, S. & Huq, A. Algorithmic decision making and the cost of fairness. *In Proceedings of the 23rd ACM SIGKDD International Conference on Knowledge Discovery and Data Mining*, 797-806 (2017). https://doi.org/10.1145/3097983.3098095

2 Feldman, M., Friedler, S. A., Moeller, J., Scheidegger, C. & Venkatasubramanian, S. Certifying and Removing Disparate Impact. *In Proceedings of the 21th ACM SIGKDD International Conference on Knowledge Discovery and Data Mining (KDD '15)*, 259-268 (2015). https://doi.org:10.1145/2783258.2783311

3 Hardt, M., Price, E., Price, E. & Srebro, N. Equality of Opportunity in Supervised Learning. *In Proceedings of the 30th International Conference on Neural Information Processing Systems* **29** (2016). https://doi.org:10.5555/3157382.3157469

4 Agarwal, A., Beygelzimer, A., Dudik, M., Langford, J. & Wallach, H. A Reductions Approach to Fair Classification. *In Proceedings of the 35th International Conference on Machine Learning*, 60-69 (2018).

5 Berk, R., Heidari, H., Jabbari, S., Kearns, M. & Roth, A. Fairness in Criminal Justice Risk Assessments: The State of the Art. *Sociological Methods & Research* **50**, 3-44 (2021). https://doi.org:10.1177/0049124118782533

6 Cui, S. *et al.* Towards Model-Agnostic Post-Hoc Adjustment for Balancing Ranking Fairness and Algorithm Utility. *In Proceedings of the 27th ACM SIGKDD Conference on Knowledge Discovery & Data Mining*, 207-217 (2021). https://doi.org:10.1145/3447548.3467251

7 Dwork, C., Hardt, M., Pitassi, T., Reingold, O. & Zemel, R. Fairness through Awareness. *In Proceedings of the 3rd Innovations in Theoretical Computer Science Conference*, 214–226 (2012). https://doi.org:10.1145/2090236.2090255

8 Kusner, M., Loftus, J., Russell, C. & Silva, R. Counterfactual fairness. *In Proceedings of the Twenty-Eighth International Joint Conference on Artificial Intelligence (IJCAI-19)* **30**, 4067-4077 (2017). https://doi.org:https://doi.org/10.24963/ijcai.2019/199

9 Censor, Y. Pareto optimality in multiobjective problems. *Appl Math Optim* **4**, 41-59 (1977). https://doi.org:10.1007/BF01442131

10 Moulin, H. *Fair Division and Collective Welfare*. (2003).

11 Ali Meerza, S. I., Li, Z., Liu, L., Zhang, J. & Liu, J. Fair and Privacy-Preserving Alzheimer's Disease Diagnosis Based on Spontaneous Speech Analysis via Federated Learning. *Annual International Conference of the IEEE Engineering in Medicine and Biology Society. IEEE Engineering in Medicine and Biology Society. Annual International Conference* **2022**, 1362-1365 (2022). https://doi.org:10.1109/EMBC48229.2022.9871204

12 Lyu, L., Li, Y., Nandakumar, K., Yu, J. & Ma, X. How to Democratise and Protect AI: Fair and Differentially Private Decentralised Deep Learning. *IEEE Transactions on Dependable and Secure Computing* **19**, 1003-1017 (2022). https://doi.org:10.1109/TDSC.2020.3006287

13 Kamiran, F. & Calders, T. Data preprocessing techniques for classification without discrimination. *Knowl Inf Syst* **33**, 1-33 (2012). https://doi.org:10.1007/s10115-011-0463-8

14 Puyol-Antón, E. *et al.* Fairness in Cardiac MR Image Analysis: An Investigation of Bias Due to Data Imbalance in Deep Learning Based Segmentation. *Medical Image Computing and Computer Assisted Intervention – MICCAI 2021: 24th International Conference, Strasbourg, France, September 27–October 1, 2021, Proceedings, Part III*, 413–423 (2021). https://doi.org:10.1007/978-3-030-87199-4_39

15 Wei, D., Ramamurthy, K. N. & Calmon, F. P. Optimized score transformation for consistent fair classification. *In Proceedings of the 23rd International Conference on Artificial Intelligence and Statistics* **22**, 1673-1683 (2021).

16 Madras, D., Creager, E., Pitassi, T. & Zemel, R. Fairness through Causal Awareness: Learning Causal Latent-Variable Models for Biased Data. *In Proceedings of the Conference on Fairness, Accountability, and Transparency*, 349-358 (2019). https://doi.org:10.1145/3287560.3287564

17 Zhao, Q., Adeli, E. & Pohl, K. M. Training confounder-free deep learning models for medical applications. *Nature Communications* **11** (2020). https://doi.org:10.1038/s41467-020-19784-9

18 Kamiran, F. & Calders, T. Classifying without discriminating. *In Proceedings of Control and Communication 2009 2nd International Conference on Computer*, 1-6 (2009). https://doi.org:10.1109/IC4.2009.4909197

19 Liu, X. J. *et al.* Projection-Wise Disentangling for Fair and Interpretable Representation Learning: Application to 3D Facial Shape Analysis. *In Proceedings of International Conference on Medical Image Computing and Computer Assisted Intervention (MICCAI)* **12905**, 814-823 (2021). https://doi.org:10.1007/978-3-030-87240-3_78

20 Thompson, H. M. *et al.* Bias and fairness assessment of a natural language processing opioid misuse classifier: Detection and mitigation of electronic health record data disadvantages across racial subgroups. *Journal of the American Medical Informatics Association* **28**, 2393-2403 (2021). https://doi.org:10.1093/jamia/ocab148

21 Rodolfa, K. T., Lamba, H. & Ghani, R. Empirical observation of negligible fairness-accuracy trade-offs in machine learning for public policy. *Nature Machine Intelligence* **3**, 896-904 (2021). https://doi.org:10.1038/s42256-021-00396-x

22 Taati, B. *et al.* Algorithmic Bias in Clinical Populations-Evaluating and Improving Facial Analysis Technology in Older Adults With Dementia. *Ieee Access* **7**, 25527-25534 (2019). https://doi.org:10.1109/access.2019.2900022
